# *NTRK* fusion events and targeted treatment of advanced radioiodine refractory thyroid cancer

**DOI:** 10.1007/s00432-023-05134-x

**Published:** 2023-08-07

**Authors:** Viktoria Florentine Koehler, Josefine Achterfeld, Natalie Sandner, Christine Koch, Jonas Paul Wiegmann, Philipp Ivanyi, Lukas Käsmann, Renate Pusch, Dominik Wolf, Mihaela Chirica, Thomas Knösel, Melanie-Christin Demes, Joerg Kumbrink, Thomas J. Vogl, Gesine Meyer, Christine Spitzweg, Joerg Bojunga, Matthias Kroiss

**Affiliations:** 1 Department of Medicine IV, LMU University Hospital, LMU Munich, Munich, Germany; 2 Department of Medicine I, Goethe University Hospital, Frankfurt am Main, Germany; 3 Department of Hematology, Hemostasis, Oncology and Stem Cell Transplantation, Hannover Medical School, Hannover, Germany; 4 Department of Radiotherapy and Radiation Oncology, University Hospital, LMU Munich, Munich, Germany; 5 German Cancer Consortium (DKTK), Partner Site Munich, Munich, Germany; 6 Department of Oncology and Hematology, Ordensklinikum Linz, Barmherzige Schwestern, Linz, Austria; 7 Department of Haematology and Oncology, Medical University Innsbruck, Innsbruck, Austria; 8 Department of Pathology, LMU Munich, Munich, Germany; 9 Senckenbergisches Institut für Pathologie, University Hospital Frankfurt, Frankfurt am Main, Germany; 10 Department of Diagnostic and Interventional Radiology, University Hospital Frankfurt, Frankfurt am Main, Germany; 11 Division of Endocrinology, Diabetes, Metabolism and Nutrition, Adjunct Academic Appointment, Mayo Clinic Rochester, Rochester, MN USA; 12 Department of Internal Medicine I, Division of Endocrinology/Diabetology, University of Würzburg, Würzburg, Germany; 13 Comprehensive Cancer Center Mainfranken, University of Würzburg, Würzburg, Germany

**Keywords:** Advanced thyroid cancer, *NTRK* fusion-positive cancer, TRK inhibitor, Larotrectinib, Outcome

## Abstract

**Purpose:**

Pathogenic fusion events involving neurotrophic receptor tyrosine kinase (NTRK) have been described in ~ 2% of differentiated thyroid cancer (DTC). The selective tropomyosin receptor kinase (TRK) inhibitors entrectinib and larotrectinib have been approved in a tumor agnostic manner based on phase 1/2 clinical trials. In a real-world setting at five referral centers, we aimed to describe the prevalence of *NTRK* gene fusions and the efficacy and safety of TRK inhibitor treatment for non-medullary, advanced thyroid cancer (TC).

**Methods:**

A total of 184 TC patients with testing for *NTRK* gene fusions were included. Progression-free survival (PFS) and overall survival (OS) probabilities were estimated using the Kaplan–Meier method in six patients with *NTRK* fusion-positive TC who underwent TRK inhibitor therapy.

**Results:**

8/184 (4%) patients harbored *NTRK* gene fusions. Six patients with radioiodine (RAI)-refractory TC harboring *NTRK1* (*n* = 4) and *NTRK3* (*n* = 2) gene fusions were treated with larotrectinib. Five patients (83%) had received ≥ 1 prior systemic therapy and one patient did not receive prior systemic therapy. All patients had morphologically progressive disease before treatment initiation. Objective response rate was 83%, including two complete remissions. Median PFS from start of TRK inhibitor treatment was 23 months (95% confidence interval [CI], 0–57.4) and median OS was not reached (NR) (95% CI, NR). Adverse events were of grade 1–3.

**Conclusion:**

The prevalence of *NTRK* gene fusions in our cohort of RAI-refractory TC is slightly higher than reported for all TC patients. Larotrectinib is an effective treatment option in the majority of *NTRK* gene fusion-positive advanced TC patients after prior systemic treatment and has a favorable safety profile.

**Supplementary Information:**

The online version contains supplementary material available at 10.1007/s00432-023-05134-x.

## Introduction

Patients with advanced, progressive thyroid cancer (TC) that is not amenable or refractory to radioiodine (RAI) therapy may require systemic treatment when palliative local therapeutic strategies or active surveillance are not an option (Haugen et al. 2016). The management of patients with locally advanced or metastatic non-medullary TC remains a therapeutic challenge. Recently, the therapeutic landscape of advanced TC has significantly changed with new targeted therapies becoming available.

Fusion events of neurotrophic receptor tyrosine kinase (NTRK) and rearranged during transfection (RET) drive tumorigenesis in certain cancers, including TC (Stransky et al. 2014; Koehler et al. 2020, 2022). Gene fusions of *NTRK* and *RET* result in an overexpression of chimeric tropomyosin receptor kinase (TRK) and RET proteins, respectively, with a constitutively active, ligand-independent downstream signaling and oncogenic potential. The reported presence of *NTRK* fusions in predominantly adult TC cohorts is low and ranges from 2.3 to 3.4% (Cancer Genome Atlas Research 2014; Lee et al. 2020; Liang et al. 2018; Solomon et al. 2020), with the highest prevalence in RAI-refractory differentiated thyroid cancer (DTC) (Ricarte-Filho et al. 2013). For patients in need of systemic treatment, the prevalence of *NTRK* fusions is not yet known.

The highly selective TRK inhibitors entrectinib and larotrectinib have been approved independent of the underlying tumor histology for patients with *NTRK* gene fusion-positive cancer in 2018 and 2019, respectively, based on phase 1/2 clinical trials (Doebele et al. 2020; Drilon et al. 2018; Hong et al. 2020). A pooled analysis of three phase 1/2 clinical trials with 159 adult and pediatric *NTRK* gene fusion-positive cancer patients treated with larotrectinib showed a remarkable objective response rate (ORR) of 79% including 24 patients (16%) with a complete response (CR) (Hong et al. 2020). In the subset of 28 patients with *NTRK* fusion-positive TC, the ORR was 75% including two patients (7%) with CR, and 29% in patients with anaplastic thyroid cancer (ATC) (Cabanillas et al. 2020).

In this retrospective study, we evaluated the prevalence of *NTRK* gene fusions in patients with non-medullary, advanced TC and described the patient characteristics, TRK inhibitor therapy practice, efficacy and treatment emergent adverse events (TEAE) in *NTRK* gene fusion-positive advanced TC patients receiving larotrectinib at three German and two Austrian tertiary care centers.

## Methods: patients

### Setting

Retrospectively collected data were obtained from records of patients diagnosed with non-medullary, advanced TC between 2002 and 2021 in three German and two Austrian tertiary care centers. Numbers for *NTRK* gene fusion testing were available from three German tertiary care centers. Data from two Austrian centers were not available. Data were collected as part of the German Study Group for Rare Tumors of the Thyroid gland approved by the ethics committee of the University of Würzburg [96/13] and subsequently by the ethics of all participating centers or by a waiver of consent, approved by the local institutional review boards for this minimal risk study. The data cut-off was 28 February 2023, and the median study follow-up was 19 months (range, 4–39).

### Diagnostic strategy for *NTRK* fusions

The presence of *NTRK* fusions in patients receiving TRK inhibitor therapy was identified by commercially available oncology genomic profiling assays, including Archer Fusionplex Lung or OncologyResearch panels (IDT, ArcherDX), Oncomine Comprehensive Assay v3 (Thermo Fisher), Oncomine Focus Assay (Thermo Fisher), TruSight Tumor 170 kit (Illumina) and TruSight Oncology 500 kit (Illumina) from primary or metastatic tissue, according to the procedures established by each laboratory (Kirchner et al. 2020; Pfarr et al. 2020).

### Data acquisition

**Fig. 1:**
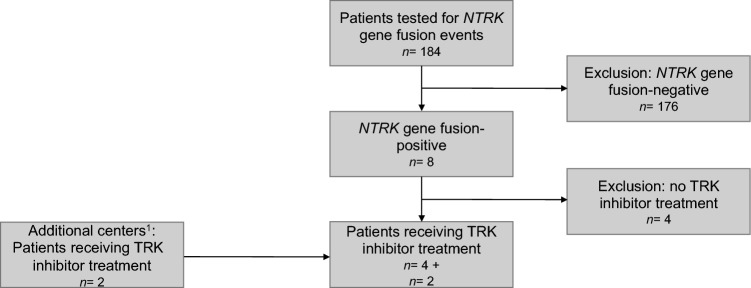
Selection of patients. Abbreviations: *NTRK* neurotrophic receptor tyrosine kinase; *TRK* tropomyosin receptor kinase. ^1^ Two Austrian centers without data on the number of cases tested

Eligible patients were adults with advanced non-medullary TC receiving *NTRK* gene fusion event testing. Parameters reflecting TRK inhibitor therapy practice were collected in patients with molecular evidence of *NTRK* fusion-positive advanced TC who underwent TRK inhibitor therapy with a specific TRK inhibitor outside of a clinical trial. The selection of patients is shown in Fig. 1. Treatment and follow-up were done according to local clinical practice of participating centers. Response was assessed locally according to standard of care by 2-deoxy-2-[fluorine-18]fluoro-D-glucose integrated with CT (^18^F-FDG PET/CT), CT, magnetic resonance imaging (MRI) of the liver and bone scintigraphy and with serum thyroglobulin (Tg) testing every 3–6 months. Bone metastases were not considered as target lesions, except for new occurrence of bone metastases on treatment. Patients still alive were censored at last follow-up.

### Statistical analysis

For categorical variables, we calculated relative and absolute frequencies; for numeric variables, we calculated medians and ranges. Progression-free survival (PFS) and overall survival (OS) probabilities were estimated using the Kaplan–Meier method. Microsoft Office Excel Version 16.55 was used for graphical presentation and additional analyses. Statistical analyses were performed with SPSS Version 26 (IBM, Chicago, IL, USA).

## Results

### *NTRK* gene fusion testing

In three centers with numerical data available, 184 patients with non-medullary, advanced TC were tested for the presence of *NTRK* gene fusions between 2018 and 2022. Overall, 8/184 (4%) were *NTRK* fusion positive, four patients required a systemic treatment and started TRK inhibitor treatment with larotrectinib. Two patients receiving TRK inhibitor treatment were included from two Austrian centers without data on the number of cases tested.

The analysis included patients with advanced TC with the following histological types based on the 2022 WHO classification of thyroid neoplasms (Baloch et al. 2022): 73 papillary thyroid cancer (PTC), 62 follicular thyroid cancer (FTC), 22 ATC, 21 poorly differentiated thyroid cancer (PDTC), and 6 oncocytic TC. Supplementary Table 1 shows *NTRK* gene fusion partners of all patients with *NTRK* gene fusions.

### Characteristics of patients with TRK inhibitor treatment

Baseline clinical characteristics of the six treated patients (5 females, 1 male), all of whom received larotrectinib, are shown in Table 1. Median follow-up from initial TC diagnosis was 7 years (range, 1–18) and median follow-up from start of larotrectinib therapy 19 months (range, 4–39). *NTRK* fusions discovered involved TRKA (*NTRK1*) in 4 patients (67%), and TRKC (*NTRK3*) in 2 patients (33%) with 5 unique fusion partners in primary (3/6, 50%) or metastatic tumor tissue (3/6, 50%).

**Table 1:** Patient characteristics of the study cohort

Patient characteristics	Number of patients (%; total number)
Number of patients	6
Female sex	5 (83)
Median age at first diagnosis (years) (range)	61 (31–70)
Tumor entity	
PTC	4 (67)
PDTC	1 (17)
ATC	1 (17)
*NTRK* gene fusion event	
NTRK1	4 (67)
NTRK2	0
NTRK3	2 (33)
UICC stage at first diagnosis	
II	1 (17)
III	0
IV	5 (83)
Lymphatic metastasis at first diagnosis	5 (83)
Distant metastasis at first diagnosis	2 (40%; n = 5)
Brain	0
Lung	2 (100)
Liver	1 (50)
Mediastinum	0
Bone	1 (50)

Abbreviations: *PTC* papillary thyroid cancer; *PDTC* poorly differentiated thyroid cancer; *ATC* anaplastic thyroid cancer; *NTRK* neurotrophic receptor tyrosine kinase; *UICC* Union for International Cancer Control

### Tumor-specific therapy

Five patients (83%) underwent total thyroidectomy and RAI therapy as initial therapy. One patient (17%) received hemithyroidectomy. The patient with ATC (17%) did not receive RAI therapy. All patients had RAI-refractory disease by the time of study inclusion. Before larotrectinib therapy, 4 patients (67%) received multi-tyrosine kinase inhibitor (MKI) treatment with sorafenib and/or lenvatinib. Two patients (50%) received one MKI (lenvatinib) and 2 patients (50%) received two MKIs (sorafenib followed by lenvatinib) before larotrectinib treatment. One patient (17%) received selitrectinib (LOXO-195) on a “named-patient basis”, a next-generation pan-TRK inhibitor after treatment with larotrectinib. Treatment for local recurrence before larotrectinib initiation was surgery in 2 patients, and external beam radiation of the neck in 4 patients. Treatment of distant metastases included surgery in 5, and external beam radiation in 2 patients. One patient did not receive systemic treatment, treatment for local recurrence or treatment of distant metastases before study inclusion.

Treatment characteristics and response to larotrectinib treatment are summarized in Table 2. Median age at larotrectinib initiation was 69 years (range,  49–74) and median time between initial diagnosis and treatment initiation was 49 months (range, 12–209). Indication for TRK inhibitor treatment was progressive disease (PD) during MKI treatment in 4 patients, PD during treatment with another TRK inhibitor in 1 patient and PD in metastatic RAI-refractory DTC without prior systemic treatment in 1 patient. At the time of larotrectinib initiation, 4 patients had local regional lymph node metastases, and 6 distant metastases (brain 1 [17%], mediastinal lymph nodes 2 [33%], lung 5 [83%], liver 2 [33%], pleura 2 [33%], and osteolytic bone metastases 3 [50%]). All patients with bone metastases received anti-resorptive therapy (ART). Two (67%) patients received bisphosphonates, and 1 (33%) patient was sequentially treated with bisphosphonates and denosumab.

**Table 2:** TRK inhibitor treatment characteristics and response rates

	TRK inhibitor treatment
Number of patients (%)	6 (100)
Median duration of treatment (range), in months	15 (4–30)
Receiving full dose of 100 mg BID at the beginning, no. of patients (%)	6 (100)
Dose reduction required, no. of patients (%)	1 (17)
ORR	83
Best response	
CR, no. of patients (%)	2 (33)
PR, no. of patients (%)	3 (50)
SD ≥ 24 weeks, no. of patients (%)	0
Median PFS, in months, (95% CI)	23 (0–57.4)
Median OS, in months, (95% CI)	NR (NR)
6-month survival rate, in %	100
12-month survival rate, in %	100

Abbreviations: *TRK* tropomyosin receptor kinase; *BID* bis in die; *CR* complete response; *PR* partial remission; *ORR* objective response rate; *SD* stable disease; *PFS* progression-free survival; 95% CI, 95% confidence interval; NR, not reached; OS, overall survival

### Efficacy of larotrectinib treatment

**Fig. 2:**
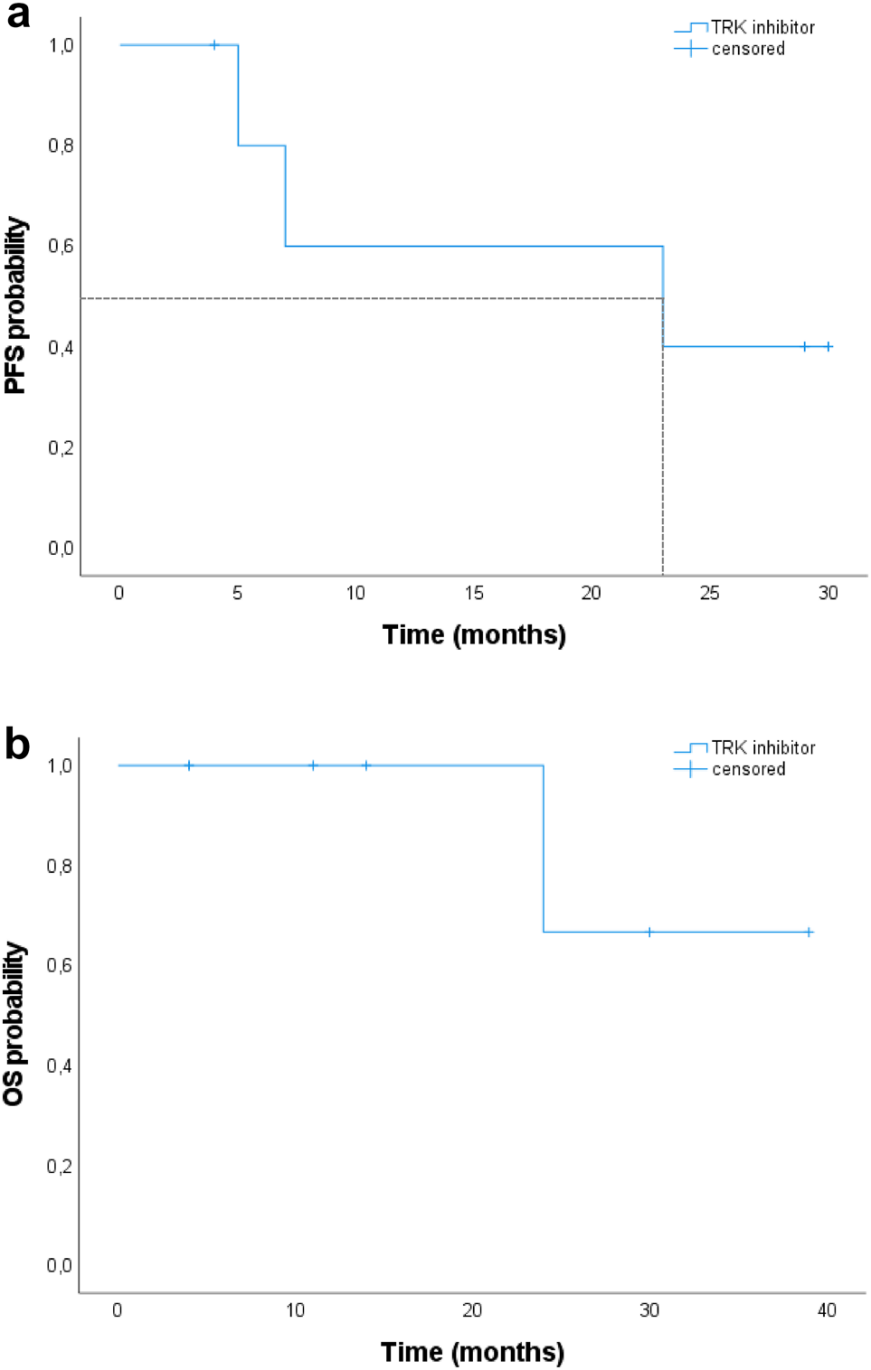
PFS and OS in months in patients receiving TRK inhibitor therapy (*n* = 6) **a** PFS in patients receiving TRK inhibitor therapy. **b** OS in patients receiving TRK inhibitor therapy. Abbreviations: *TRK* tropomyosin receptor kinase; *PFS* progression-free survival; *OS* overall survival

The median PFS from start of TRK inhibitor treatment was 23 months (95% confidence interval [95% CI], 0–57.4; Fig. 2a), and median OS was not reached (NR) (95% CI, NR; Fig. 2b). Metastatic sites with the best morphological response were bone metastases (2 [67%]; *n* = 3), lymph nodes (2 [67%]; *n* = 3), and pulmonal metastases (2 [67%]; *n* = 3). At the data cut-off, 2 patients (33%) were still receiving TRK inhibitor treatment, 3 patients (50%) discontinued due to PD, and 1 patient (17%) discontinued due to TEAE grade 3 (gait instability with pre-existing peripheral polyneuropathy). One patient (17%) had died, and 2 patients (33%) were lost to follow-up.

### Safety and tolerability of larotrectinib

Four (67%) patients showed TEAE, of all the adverse events being of grades 1–3. One patient discontinued treatment due to TEAEs. The most frequently reported drug-related TEAE were weight gain (33%), and fatigue (33%). Further TEAEs included anemia (17%), dizziness (17%), elevated liver enzymes (17%), gait instability (17%), and polyneuropathy (17%).

### Selitrectinib treatment

One patient received the investigational TRK inhibitor selitrectinib outside of a clinical trial after treatment with larotrectinib due to PD. After 3 months of treatment and continuation of PD, the patient was switched to best supportive care. TEAE included nausea and fatigue grade 3.

### Case description

**Fig. 3:**
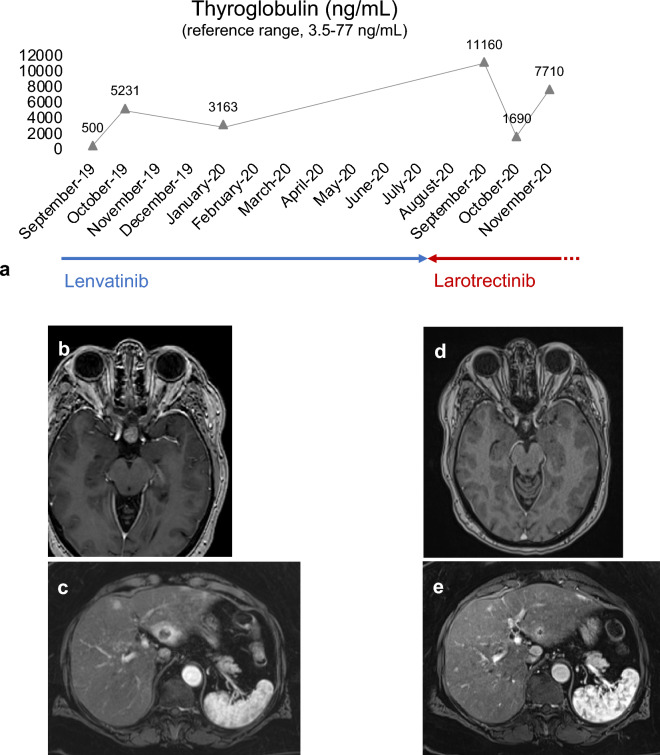
Biochemical and radiologic responses to larotrectinib **a** Tumor progression with Tg increased to 5231 ng/mL (reference range, 3.5–77 ng/mL) despite lenvatinib treatment. Further molecularly informed treatment with the TRK inhibitor larotrectinib at a dose of 100 mg BID decreased Tg to 1690 ng/mL within four weeks. The further increase of serum Tg after start of NTRK inhibitor treatment was interpreted as a functional sign of redifferentiation. As of the time of this report, at 30 months from the start of larotrectinib, the patient continues on treatment with SD and no TEAE **b** Brain MRI reveals a new pituitary metastasis during lenvatinib treatment **c** A MRI of the liver during lenvatinib treatment reveals disseminated hepatic metastatic disease **d** Significant tumor shrinkage in the course of therapy with larotrectinib **e** Radiologic follow-up revealed PR of liver metastases. *BID* bis in die

A 67-year-old female patient diagnosed initially with FTC in 11/2017 presented for follow-up of advanced FTC with metastatic sites including lymph nodes, liver and lung. Time between initial diagnosis and evidence of RAI-refractory disease was 5 months. Follow-up with ^18^F-FDG PET/CT revealed PD and an increasing serum Tg level of 5231 ng/mL (reference range, 3.5–77 ng/mL) compared to 500 ng/mL one month before. Due to PD with detection of new bone and pituitary metastases with secondary adrenal insufficiency, hydrocortisone replacement and tumor-specific treatment with lenvatinib 24 mg/d was initiated. At that time, the formalin-fixed paraffin-embedded tumor sample underwent testing with the Oncomine Comprehensive Assay v3. The analysis revealed a SQSTM1/NTRK3 gene fusion and reference pathology led to the revised diagnosis of oncocytic TC with poorly differentiated features. After 7 months of lenvatinib, treatment was switched to larotrectinib 100 mg twice daily due to PD and increasing Tg levels of 11,160 ng/mL. First follow-up revealed partial remission (PR). Biochemical and radiologic response are summarized in Fig. 3.

## Discussion

Here, we studied the prevalence of *NTRK* gene fusion events in TC patients requiring systemic therapy and report real-world clinical data from patients with *NTRK* gene fusion-positive non-medullary, advanced TC who received treatment with larotrectinib outside of a clinical trial.

*NTRK* fusions in TC are rare with a reported frequency of about 2% (Cancer Genome Atlas Research 2014; Rosen et al. 2020). The slightly higher frequency of 4% in our study cohort is most likely due to selection bias. Nevertheless, the actual frequency of *NTRK* fusions in TC could be underestimated due to limitations of currently available testing modalities (Solomon et al. 2020). Therefore, a recent multicentric study tested the comparative performance of different methodologies regarding their ability to detect *NTRK* gene fusions and suggested a test algorithm based on entity (Pfarr et al. 2020). Moreover, in recent years, multinational ring trials for *NTRK* fusion detection were available to test the suitability of the, respectively, used method in the participating laboratories (Kirchner et al. 2020).

The female predominance of 83% in our treated cohort is consistent with the data from Chu et al*.*, who characterized 11 *NTRK*-rearranged TC by clinicopathologic and molecular features and observed a female predominance of 73% (Chu et al. 2020). Furthermore, they observed *NTRK*-rearrangement only in tumors which had been originally diagnosed as PTC (Chu et al. 2020). In comparison, the series by Cabanillas et al., Fazeli et al*.,* and Park et al*.* comprises patients with ATC/FTC, ATC/PDTC, and PTC/PDTC/ATC, respectively (Cabanillas et al. 2020; Fazeli et al. 2020; Park et al. 2022). The proportion of different histologic subtypes in our complete *NTRK* fusion-positive cohort of 10 patients is in accordance with the published data, confirming that *NTRK* fusions occur predominantly in PTC, but with a lesser frequency also in less differentiated tumors.

Among the treated patients, the frequency of distant metastases at initial diagnosis (40%) is comparable to the data from Chu et al. and Fazeli et al*.*, reporting distant metastases in about 25% and 38%, respectively (Chu et al. 2020; Fazeli et al. 2020). The rate of NTRK1 fusions in our cohort of 10 patients is higher at 60% compared to the recently published series (Cabanillas et al. 2020; Chu et al. 2020; Fazeli et al. 2020; Park et al. 2022). Though, all PDTC and ATC patients showed a *NTRK3* fusion which is in accordance with reported results by Fazeli et al*.* showing the *NTRK3* fusion to be the most common *NTRK* fusion in ATC and PDTC patients (7/8, 87%) (Fazeli et al. 2020). No case showed a *NTRK2* fusion. This is consistent with published data from non-medullary TC patients (Cabanillas et al. 2020; Chu et al. 2020; Fazeli et al. 2020), except for the recently published case series, which showed a *NTRK2* fusion in 1/4 of the patients (Park et al. 2022).

The efficacy of larotrectinib in our retrospective analysis is in accordance with previously reported results; the ORR was 83% versus 75% across various *TRK* fusion-positive tumor types in a primary analysis set of 55 patients in a phase 1/2 basket trial and 79% in a pooled analysis of three phase 1/2 clinical trials (Drilon et al. 2018; Hong et al. 2020). Data from patients with locally advanced or metastatic *NTRK* fusion-positive TC pooled from two larotrectinib clinical trials published in abstract form showed an ORR of 75% including patients with ATC, and 90% only in patients with DTC (Cabanillas et al. 2020). We found CR in 33%, and PR in 50%, which translated into a PFS of 23 months. Our case report of a patient with oncocytic TC and evidence of poorly differentiated features confirms that druggable fusion events may be present in oncocytic TC with similar responsiveness to TRK inhibitor therapy.

No patient showed primary resistance to larotrectinib, defined as a best response of PD (Drilon et al. 2018). Acquired resistance to larotrectinib, defined as PD after objective response or SD for at least 6 months (Drilon et al. 2018; Jackman et al. 2010), was observed in 1 patient (16%). The patient underwent repeated testing to determine the mechanisms of acquired resistance to larotrectinib. Histopathology of a soft tissue sample of the neck revealed avital squamous keratinized epithelium with evidence of squamous cell carcinoma. A second next generation sequencing (NGS) performed did not exhibit the same mutation profile as the initial tissue sample of a lung metastasis: the presence of a *NTRK1* fusion was not confirmed, potentially explaining the lack of response in this patient. Whether the histopathological result represents squamous cell differentiation at metastatic site before or during treatment with larotrectinib or involvement by concomitant squamous cell carcinoma from another organ remains a matter of debate. Furthermore, no known larotrectinib-resistant mutation was detected. Drilon et al. found acquired resistance to larotrectinib in 2 patients: repeated testing after PD revealed mutations in the kinase domain affecting the *NTRK* gene involved in the fusion (Drilon et al. 2017). A next-generation TRK inhibitor can potentially overcome acquired resistance to earlier-generation TRK inhibitors (Drilon et al. 2017).

Based on recent evidence long-term administration of larotrectinib appears feasible (Hong et al. 2020). Maximum treatment duration in our cohort was 30 months and two patients are still receiving treatment with larotrectinib at data cut-off. The favorable safety and tolerability profile in our cohort is consistent with the published data without grade 4/5 adverse events in our cohort (Drilon et al. 2018).

Our study of six *NTRK* fusion-positive advanced TC patients treated with larotrectinib at five specialized centers in Germany and Austria has some limitations: missing data due to its retrospective nature, small patient number, lack of systematic follow-up, heterogeneity of patient management, and the evaluation of imagings by different radiologists.

## Conclusion

We demonstrate that larotrectinib, a highly selective TRK inhibitor, is an effective drug in patients with locally advanced or metastatic *NTRK* fusion-positive TC, regardless of the therapy line. Additional real-world data from a larger cohort of TC patients with a longer follow-up are needed, as well as data comparing larotrectinib to standard of care.

### Supplementary Information

Below is the link to the electronic supplementary material.

Supplementary file1 (DOCX 18 KB)

## Data Availability

The datasets generated during and/or analyzed during the current study are available from the corresponding author on reasonable request.
