# Supplementary material for: NTRK fusion events and targeted treatment of advanced radioiodine refractory thyroid cancer
Source: J Cancer Res Clin Oncol. 2023 Aug 7;149(15):14035–43. doi: 10.1007/s00432-023-05134-x (PMC10590332; doi:10.1007/s00432-023-05134-x)
Supplement: Supplementary file 1 — Supplementary file1 (DOCX 18 KB) [file 432_2023_5134_MOESM1_ESM.docx]

### Supplementary Table 1 *NTRK* fusions

| *NTRK gene* | *Gene fusion* | *Break points* | *Exon* | *refseq* | *Fusion partner* | *Tumor entity* | *TRK inhibitor treatment* |
| --- | --- | --- | --- | --- | --- | --- | --- |
| NTRK1 | SQSTM1::NTRK1 | chr5:179252226 chr1:15684436 | 5 10 | NM_001142299.2 NM_001007792.1 | SQSTM1 | PTC |  |
|  | TPR::NTRK1 | chr1:186337017  chr1:156845310 | 4  12 | NM_003292.3  NM_002529.3 | TPR | PTC | Larotrectinib |
|  | TPR::NTRK1 | chr1:186319355  chr1:156845312 | 21  12 | NM_003292.3  NM_002529.3 | TPR | PTC | Larotrectinib |
|  | TPM3::NTRK1 | chr1:154142876  chr1:156844363 | 7  10 | NM_001007792.1  NM_153649.3 | TPM3 | PTC | Larotrectinib |
|  | TPM3::NTRK1 | chr1:154142878 chr1:156845312 | 8 12 | NM_152263.4  NM_001007792.1 | TPM3 | PTC | Larotrectinib |
|  | TPM3::NTRK1 | chr1:154142876  chr1:156844363 | 4  10 | NM_152263.3  NM_002529.3 | TPM3 | PTC |  |
| NTRK3 | ETV6::NTRK3 | chr12:12006495  chr15:88576276 | 4  14 | NM_001987.5  NM_002530.4 | ETV6 | ATC | Larotrectinib |
|  | ETV6::NTRK3 | chr12:12006495 chr15:88576276 | 4  14 | NM_001987.4  NM_002530 | ETV6 | PTC |  |
|  | ETV6::NTRK3 | chr12:12006495 chr15:88576276 | 4  14 | NM_001987.4  NM_002530 | ETV6 | PTC |  |
|  | SQSTM1::NTRK3 | chr5:179252226 chr15:88576276 | 5  14 | NM_003900.5  NM_002530 | SQSTM1 | PDTC | Larotrectinib |

Abbreviations: NTRK, neurotrophic receptor tyrosine kinase; UICC, Union for International Cancer Control; TRK, tropomyosin receptor kinase; PTC, papillary thyroid cancer; ATC, anaplastic thyroid cancer; PDTC, poorly differentiated thyroid cancer
